# Histological correlates of hippocampal magnetization transfer images in drug-resistant temporal lobe epilepsy patients

**DOI:** 10.1016/j.nicl.2020.102463

**Published:** 2020-10-08

**Authors:** Jose Eduardo Peixoto-Santos, Tonicarlo R. Velasco, Carlos Gilberto Carlotti, Joao Alberto Assirati, Gustavo Henrique de Souza e Rezende, Katja Kobow, Roland Coras, Ingmar Blümcke, Carlos Ernesto Garrido Salmon, Antonio Carlos dos Santos, Joao Pereira Leite

**Affiliations:** a Department of Neurology and Neurosurgery, Paulista Medical School, UNIFESP, Sao Paulo, Brazil; b Department of Neurosciences and Behavioral Sciences, Ribeirao Preto Medical School, University of Sao Paulo, Ribeirao Preto, Brazil; c Department of Surgery and Anatomy, Ribeirao Preto Medical School, University of Sao Paulo, Ribeirao Preto, Brazil; d Center for Technology and Research in Magneto-Resonance (CTPMAG), Department of Physiology and Biophysics, Federal University of Minas Gerais, Belo Horizonte, Brazil; e Department of Neuropathology, University Hospital Erlangen, Erlangen, Germany; f Department of Physics and Mathematics, Faculty of Philosophy, Science and Languages of Ribeirao Preto, University of Sao Paulo, Ribeirao Preto, Brazil; g Department of Internal Medicine, Ribeirao Preto Medical School, University of Sao Paulo, Ribeirao Preto, Brazil

**Keywords:** ANOVA, Analysis of variance, CA, Cornu Ammonis, CSPG, Chondroitin sulfate proteoglycan, GFAP, Glial fibrillary acid protein, HLA-DR, Human leukocyte antigen - DR isotype, HR, Histological control, HS, Hippocampal sclerosis, ILAE, International League Against Epilepsy, IPI, Initial precipitating injury, MRI, Magnetic resonance imaging, MT, Magnetization transfer, MTR, Magnetization transfer ratio, NeuN, Neuronal nuclei, NIH, National Institutes of Health, RC, Radiological control, TLE, Temporal lobe epilepsy, VIF, Variance inflation factor, Temporal lobe epilepsy, Hippocampal sclerosis, Magnetization transfer ratio, Extracellular matrix, Neuron density

## Abstract

**Objective:**

Temporal lobe epilepsy patients (TLE) often present with hippocampal atrophy, increased T2 relaxation, and reduced magnetization transfer ratio (MTR) in magnetic resonance images (MRI). The histological correlates of the reduced hippocampal MTR are so far unknown. Since MTR is dependent on the tissue’s macromolecules, our aim was to evaluate the correlations between cellular populations, extracellular matrix molecules and the MTR in TLE patients.

**Methods:**

Patients with TLE (n = 26) and voluntaries (=20) were scanned in a 3 Tesla MRI scanner, and MTR images were calculated from 3DT1 sequences with magnetization pulse *on resonance*. Immunohistochemistry for neurons, reactive astrocytes, activated microglia, and extracellular matrix chondroitin sulfate were performed in formalin fixed, paraffin embedded tissues of TLE and autopsy controls (n = 10). Results were considered significant with adjusted p < 0.05.

**Results:**

Compared to the respective controls, TLE patients had reduced hippocampal MTR, increased reactive astrocytes and activated microglia, increased extracellular chondroitin sulfate, and reduced neuron density, compares to controls. MTR correlated positively with neuron density in CA3 and with chondroitin sulfate in CA3 and CA1. Multiple linear regressions reinforced the correlations between chondroitin sulfate and MTR.

**Significance:**

Our data indicate that extracellular matrix molecules are the most significant histological correlates of magnetization transfer ratio in the hippocampus of TLE patients.

## Highlights

• Hippocampal MTR in TLE does not add to volume or T2 signal regarding lateralization.
• Extracellular CSPG are the most relevant correlate to MTR in hippocampal sclerosis.
• Increased CSPG counterbalances neuron loss over the reduction in hippocampal MTR.

## 1 Introduction

Hippocampal sclerosis (HS) is the most common pathological finding in adults with drug-resistant epilepsy (Blumcke et al., 2017). In the pathological evaluation, HS is characterized by differential neuron loss, often more severe in CA1 and CA4 (Blumcke et al., 2013). Besides neuron loss, gliosis and changes in extracellular matrix (ECM) proteins are seen in the sclerotic hippocampus of these patients (Crespel et al., 2002, Van Paesschen et al., 1997, Perosa et al., 2002, Peixoto-Santos et al., 2015). Moreover, gliosis can even be seen in the hippocampi of temporal lobe epilepsy patients (TLE) without neuron loss (Thom, 2014). The presurgical magnetic resonance imaging (MRI) of these patients often shows hippocampal volume loss and increased T2 signal (Berkovic et al., 1991, Briellmann et al., 2002, Cendes et al., 1993). However, in up to 15% of the TLE patients, no abnormalities are observed in the standard MR evaluation (Bernasconi et al., 2000, Jackson et al., 1994). Although the normal MRI patients often have only milder or no neuron loss, some can have neuron loss and other pathological changes as severe as those cases with hippocampal sclerosis detected on MRI (Peixoto-Santos et al., 2015, Jackson et al., 1994). Since the presence of HS is often an indicator of good surgical outcome in TLE (Blumcke et al., 2013, Thom, 2014), it is crucial to improve the pre-surgical detection of those MRI-negative cases with pathologically proven HS, as well as more subtle pathological changes in the hippocampus.

New quantitative MRI techniques could improve the detection of more subtle abnormalities seen in epilepsy patients (Diniz et al., 2011), especially in those cases were quantitative measurements in the more traditional T1- and T2-weighted images are subtle or absent. Magnetic transfer ratio (MTR), an imaging protocol that evaluates macromolecules not visible in either T1-weighted or T2-weighted images, is often reduced in regions with EEG abnormalities (Rugg-Gunn et al., 2003). While some studies have indicated the association between myelin loss and MTR reduction in the white matter, and between neuron loss and MTR reduction in the cerebral cortex, there remains to be determined which pathological changes are responsible for the decreased hippocampal MTR in the hippocampus of TLE patients. We previously found that ECM chondroitin sulfate proteoglycan (CSPG) could impact both hippocampal volume (Peixoto-Santos et al., 2015) and T2 signal relaxation time (Peixoto-Santos et al., 2017). Since MTR is influenced by the macromolecules present in the tissue, our objective was to evaluate the correlations between the MTR and chondroitin sulfate, as well as with cellular populations, in the hippocampus of drug-resistant temporal lobe epilepsy patients.

## 2 Materials and Methods

### 2.1 Patients

Twenty-six drug-resistant TLE patients were selected during the presurgical evaluation at the Epilepsy Surgery Centre (CIREP) of Ribeirao Preto Medical School. Presurgical workup included history review, neurological evaluation, neuropsychiatric memory tests, video-EEG, and an optimized MRI protocol for TLE, for the definition of epileptogenic focus. Patients with undoubted seizure focus underwent standard *en bloc* temporal lobe resection.

Thirty age-matched control cases consisted on: twenty healthy volunteers that underwent the same presurgical MRI protocol used for TLE cases (radiological controls, RC), used for defining MRI differences in TLE; ten autopsy cases (histological controls, HC) whose hippocampi were collected to serve as a standard for immunohistochemistry analysis.

Inclusion criteria were: age between 20 and 60 years for all groups; diagnosis of drug-resistant TLE (for TLE group); quantitative MRI protocol (for TLE and RC). Exclusion criteria were: the presence of MRI abnormalities (for RC); generalized or extratemporal EEG spikes for TLE (Cendes et al., 1996, Cendes et al., 2000); the presence of pathological changes in the hippocampus in histological evaluation (for HC); the presence of other brain pathology than hippocampal sclerosis (for TLE cases); postmortem time superior to 12 h (for HC; this cutoff is based on previous study showing stable protein expression up to 12 h (Peixoto-Santos et al., 2012).

This study was registered in the Brazilian's Health Ministry and approved by the local Research Ethics Committee of the Hospital das Clinicas (HCRP # 7200/2016). A written Informed Consent Term, previously approved by the Research Ethics Committee, was obtained from all patients or next-of-kin enrolled in this study, following the Declaration of Helsinki.

### 2.2 MRI protocol

All TLE cases and RC volunteers underwent MRI in a Philips Achieva 3.0 T X-series with an 8 elements phase-array head coil. For the definition of hippocampal atrophy, 3D single shot T1-weighted images were performed (TE = 3.2 ms; TR = 7 ms; flip angle = 8°; inversion pulse = 900 ms; shot interval = 2500 ms; voxel size = 1 mm^3^; FOV = 240x240 mm; acquisition time = 4.5 min). T2 relaxation was additionally measured with 2D turbo spin echo sequences (TEs = 20, 40, 60, 80, 100 ms; TR = 3000 ms; flip angle = 90°; EPI factor = 5; voxel size = 1x1x3 mm; FOV = 240x180 mm; acquisition time = 4 min). Magnetization transfer ratio was evaluated with two 3D sequences with minimal T1 and T2 weighting (TFE = 3; TE = 3 ms; TR = 3.6 ms; flip angle = 8°; magnetization transfer saturation pulse *on resonance*; voxel = 1x1x3 mm; FOV = 240x180, acquisition time = 5 min).

The hippocampus was previously segmented using FreeSurfer package (v. 5.3) and 3D-T1w images. The default command recon-all was used for the automatic segmentation of subcortical structures. The segmented hippocampal label was visually double-checked to avoid CSF contamination. The corrected label was used for calculating whole hippocampal volume in the T1 images and was also overlap in the coregistered MTR and T2 maps (calculated with homemade scripts) for extraction of whole hippocampal MTR and T2 values. Absolute hippocampal volume was considered low if below 2.5 cm^3^, similar to previous studies of our group (Peixoto-Santos et al., 2015), while T2 relaxation was considered abnormal if above average (x̅) + 2 standard deviations (s) of controls (i.e., 115.8 ms; controls = 104.7 ± 5.5 ms) and MTR was abnormal if below x̅ − 2 s of controls (i.e., 48.6%; controls = 50.4 ± 0.9%).

To compare the quantitative results with the visual diagnosis made by expert radiologists (Table 1), we calculated the asymmetry between the ipsilateral and contralateral hippocampus (ipsilateral/contralateral) and compared it with control values. Control asymmetry was defined by randomly assigning either left or right hippocampus as “ipsilateral” and “contralateral”, calculating the ipsilateral/contralateral ratio and defining the cutoff as x̅ − 2 s for volume and MTR or x̅ + 2 s for relaxation time. Thus, cutoffs were 0.85 for volume, 0.98 for MTR, and 1.09 for relaxation time. We also compared asymmetry based on a fixed 5% difference between ipsilateral and contralateral.

**Table 1:** Quantitative and qualitative MRI evaluation on all cases.

Case	Visual Analysis of the hippocampus	Quantitative evaluation of the hippocampus									HS type in histology	Outcome
		Ipsilateral			Contralateral			Asymmetry				
		Volume	T2 relaxation	MTR	Volume	T2 relaxation	MTR	Volume	Relaxation	MTR		
1	Asymmetry, with ipsilateral volume reduction and long TR hypersignal	2.30	138.79	48.31	3.00	115.03	52.82	0.77	1.21	0.91	HS1	ILAE1
2	Asymmetry, with ipsilateral volume reduction and long TR hypersignal	3.14	86.80	48.07	3.82	80.73	49.14	0.82	1.08	0.98	HS1	ILAE1
3	Asymmetry, with ipsilateral volume reduction and long TR hypersignal	2.10	97.58	44.54	3.10	88.60	45.77	0.68	1.10	0.97	HS1	ILAE2
4	Asymmetry, with ipsilateral volume reduction and long TR hypersignal	2.50	108.21	50.22	4.00	100.20	50.87	0.63	1.08	0.99	HS1	ILAE2
5	Asymmetry, with ipsilateral volume reduction and long TR hypersignal	1.50	131.97	40.69	3.20	116.49	41.34	0.47	1.13	0.98	HS1	ILAE1
6	Asymmetry, with ipsilateral volume reduction and long TR hypersignal	2.84	119.80	48.46	3.94	114.32	50.08	0.72	1.05	0.97	HS1	ILAE1
7	Asymmetry, with long TR hypersignal and loss of internal structure	3.58	124.00	40.92	3.87	94.75	37.62	0.92	1.31	1.09	HS1	ILAE3
8	Asymmetry, with ipsilateral volume reduction, loss of internal structure, and discrete long TR hypersignal	1.94	154.27	42.30	3.98	127.87	46.36	0.49	1.21	0.91	HS1	ILAE3
9	Asymmetry, with ipsilateral volume reduction and long TR hypersignal	3.63	106.03	49.68	4.41	98.78	50.79	0.82	1.07	0.98	HS1	ILAE1
10	Asymmetry, with ipsilateral volume reduction and discrete long TR hypersignal	2.62	131.71	47.89	3.66	109.17	49.92	0.72	1.21	0.96	HS1	ILAE4
11	Asymmetry, with ipsilateral volume reduction and long TR hypersignal	2.83	111.51	49.43	4.19	109.11	50.15	0.68	1.02	0.99	HS1	ILAE3
12	Asymmetry, with discrete ipsilateral volume reduction and discrete long TR hypersignal	3.70	105.88	50.88	4.90	102.34	50.21	0.76	1.03	1.01	HS1	ILAE1
13	Asymmetry, with ipsilateral volume reduction and discrete long TR hypersignal	2.06	150.22	46.73	3.72	136.58	47.78	0.55	1.10	0.98	HS1	ILAE2
14	Asymmetry, with ipsilateral volume reduction and long TR hypersignal	2.43	129.64	47.46	3.94	106.82	50.59	0.62	1.21	0.94	HS1	ILAE3
15	Asymmetry, with ipsilateral volume reduction and discrete long TR hypersignal	3.08	107.60	50.47	3.90	105.89	49.88	0.79	1.02	1.01	HS1	ILAE1
16	Asymmetry, with ipsilateral volume reduction and long TR hypersignal	2.53	134.03	46.91	4.75	108.19	49.98	0.53	1.24	0.94	HS1	ILAE1
17	Asymmetry, with discrete ipsilateral volume reduction and normal long TR signal	3.59	110.61	49.02	4.29	105.99	50.10	0.84	1.04	0.98	HS1	ILAE1
18	Asymmetry, with ipsilateral volume reduction and long TR hypersignal	3.99	113.50	49.43	4.78	108.54	50.72	0.83	1.05	0.97	HS1	ILAE3
19	Asymmetry, with discrete ipsilateral volume reduction and discrete long TR hypersignal in the hippocampal head	5.01	129.41	49.44	5.51	107.36	51.40	0.91	1.21	0.96	HS2	ILAE3
20	Asymmetry, with ipsilateral volume reduction and long TR hypersignal	3.69	98.11	50.74	4.08	95.52	50.71	0.90	1.03	1.00	HS2	ILAE3
21	Asymmetry, with ipsilateral volume reduction and long TR hypersignal	1.86	137.01	44.08	3.69	116.25	47.85	0.50	1.18	0.92	HS1	ILAE1
22	Asymmetry, with ipsilateral volume reduction and long TR hypersignal	2.40	132.81	49.41	3.20	121.34	49.81	0.75	1.09	0.99	HS1	ILAE1
23	Asymmetry, with discrete ipsilateral volume reduction, discrete long TR hypersignal, and loss of internal structure	3.83	124.26	48.81	5.64	111.27	50.47	0.68	1.12	0.97	HS1	ILAE1
24	Asymmetry, with ipsilateral volume reduction and long TR hypersignal	3.17	112.66	51.61	4.26	104.51	51.05	0.74	1.08	1.01	HS1	ILAE1
25	Asymmetry, with ipsilateral volume reduction and long TR hypersignal	2.98	141.83	47.79	4.10	111.20	49.65	0.73	1.28	0.96	HS1	ILAE2
26	Asymmetry, with ipsilateral volume reduction and long TR hypersignal	3.10	119.09	47.93	3.80	80.70	54.75	0.82	1.48	0.88	HS2	ILAE1

### 2.3 Immunohistochemistry protocol

Coronal sections from the hippocampal body of TLE and HC cases were fixed in formalin, dehydrated, clarified, and embedded in paraffin. Eight-micrometer-thick sections were submitted to immunohistochemistry with anti-NeuN (#MAB377, Chemicon; a neuronal marker), anti-GFAP (#M0761, Dako; a marker of reactive astrocytes), anti-HLA-DR (#M0746, Dako; a marker of activated microglia), and anti-CSPG (#C8035, Sigma; a broad-spectrum chondroitin sulfate proteoglycan marker), following protocols and dilutions previously published (Peixoto-Santos et al., 2017).

Micrographs from the regions of interest were collected with an AxioCamMR5 in an Axio Imager M1 microscope with the AxioVision 4.8.1 software (Zeiss). Illumination was maintained constant (3 V), and exposure varied from each protein (33 ms for NeuN and GFAP, 60 ms for HLA-DR, and 40 ms for CSPG), and micrographs were taken at 100x magnification for NeuN, GFAP, and CSPG, and at 200x magnification for HLA-DR. The analysis was performed with ImageJ 1.45 s software (NIH), with a semi-quantitative analysis of the immunopositive area fraction (Peixoto-Santos et al., 2017, Eriksson et al., 2007, Kandratavicius et al., 2015). The thresholds used were 154 ± 10 for GFAP, 50 ± 10 for HLA-DR, and 132 for CSPG. Neuron density was estimated following Abercrombie’s method, as described elsewhere (Peixoto-Santos et al., 2018). The TLE cases were classified according to the hippocampal sclerosis type (Blumcke et al., 2013). The regions of interest where the hippocampal subfields CA4, CA3, CA2, CA1, and subiculum, as delineated by ILAE Taskforce (Blumcke et al., 2013).

### 2.4 Statistics

For the parametric variables, ANOVA with Bonferroni *post hoc* test or Student’s *t*-test were performed, whereas Kruskal-Wallis with Dunn *post hoc* test or Mann-Whitney’s test were used for non-parametric data. Spearman’s correlation was performed to evaluate the association between histological and MR data, and multiple linear models were constructed based on the most relevant results. The association between categorical data and MTR class was explored with logistic regression followed by a χ^2^ test. All p-values were corrected for false discovery ratio (FDR), and differences were considered significant at adjusted p < 0.05.

## 3 Results

### 3.1 Clinical data

All subject enrolled in the present study were age-matched (HC = 46.9 ± 13.6 years, RC = 43.6 ± 7.6 years, and TLE = 43.2 ± 11.8 years, ANOVA). Postmortem interval was of 8.3 ± 4.1 h (ranging from 3 to 12 h), and most HC cases had heart failure as the main cause of death (50%), followed by sepsis (30%) and pneumonia (20%). TLE cases had the first seizure at 10.1 ± 10.0 years (median of 5.5 years, ranging from 0.5 to 35 years) and seizure recurrence at 18.0 ± 11.1 years (median of 17 years, ranging from 0.5 to 45 years). All remaining clinical data are described in Table 1.

### 3.2 MRI evaluation

**Fig. 1:**
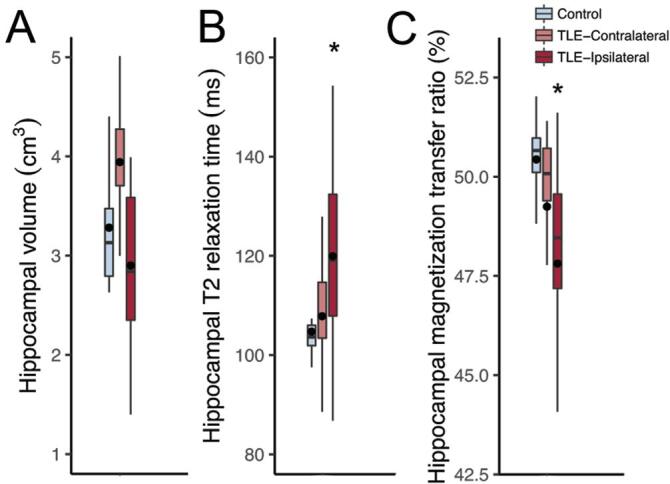
Quantitative magnetic resonance evaluation of the hippocampi from controls (radiological controls, blue boxplots) and TLE patients (red boxplots). (A) There was no difference between controls and ipsilateral (dark red boxplot) or contralateral (light red boxplot) hippocampi from TLE regarding volume. (B) Only the ipsilateral hippocampus of TLE patients presented with increased T2 relaxation time and (C) reduced magnetization transfer, when compared to controls. There was no difference between the ipsilateral and contralateral hippocampus of TLE cases. The asterisks indicate statistical difference, the line inside the boxplots indicate median and the dot indicate mean. (For interpretation of the references to colour in this figure legend, the reader is referred to the web version of this article.) (For interpretation of the references to colour in this figure legend, the reader is referred to the web version of this article.)

The automatic hippocampal analysis showed no significant difference in hippocampal volume between controls and TLE cases (Studens, p = 0.169; Fig. 1 **A**). Only nine of the TLE patients (34.6%) had hippocampal atrophy, following the cutoffs of previous quantitative studies with a different set of cases (Peixoto-Santos et al., 2015). Hippocampus from TLE patients presented with higher relaxation time than controls (Mann-Whitney, p < 0.001; Fig. 1 **B**). The hippocampi from TLE cases had a significant reduction in MTR compared to controls (Mann-Whitney, p < 0.001; Fig. 1 **C**). Based on control hippocampi average and standard deviation, cases below 95% MTR of controls (i.e., below 48.6% magnetization transfer) were classified as having low MTR, and those above as having normal MTR. In the patients without hippocampal volume loss, the low MTR was seen in 50% of cases. As for the cases with hippocampal atrophy, 88% had low MTR. The combination with T2 relaxation increases the detection of the abnormal hippocampus to 64% of normal volume cases. Finally, from all cases, two presented only MTR reduction, and eight were MRI-negative patients (i.e., normal hippocampal volume, T2 relaxation, and MTR).

Qualitative (visual) evaluation of expert radiologists indicated marked asymmetric hippocampal volume in 84% of the cases, discrete volume asymmetry in 12% and normal volume in 4%. Visual T2-weighted and FLAIR signal were considered markedly asymmetric in 69% of the cases, discretely increased in 27%, and normal in 4%. With the combination of qualitative volume and signal evaluation, all subjects had some hippocampal asymmetry (see Table 1). In a similar approach, the quantitative data matched the visual assessment. Based on the controls, the asymmetry indicated that the epileptogenic (ipsilateral) hippocampus was smaller in 88% of the cases, had increased T2 relaxation in 54% % of the patients, and MTR was lower in 50% of the cases. However, the difference in MTR was more subtle, and a difference higher than 5% was only seen in 23% of the cases (versus 100% with volume and 81% with T2 relaxation differences higher than 5% of the contralateral value; Table 1).

### 3.3 Cellular populations and extracellular matrix

**Fig. 2:**
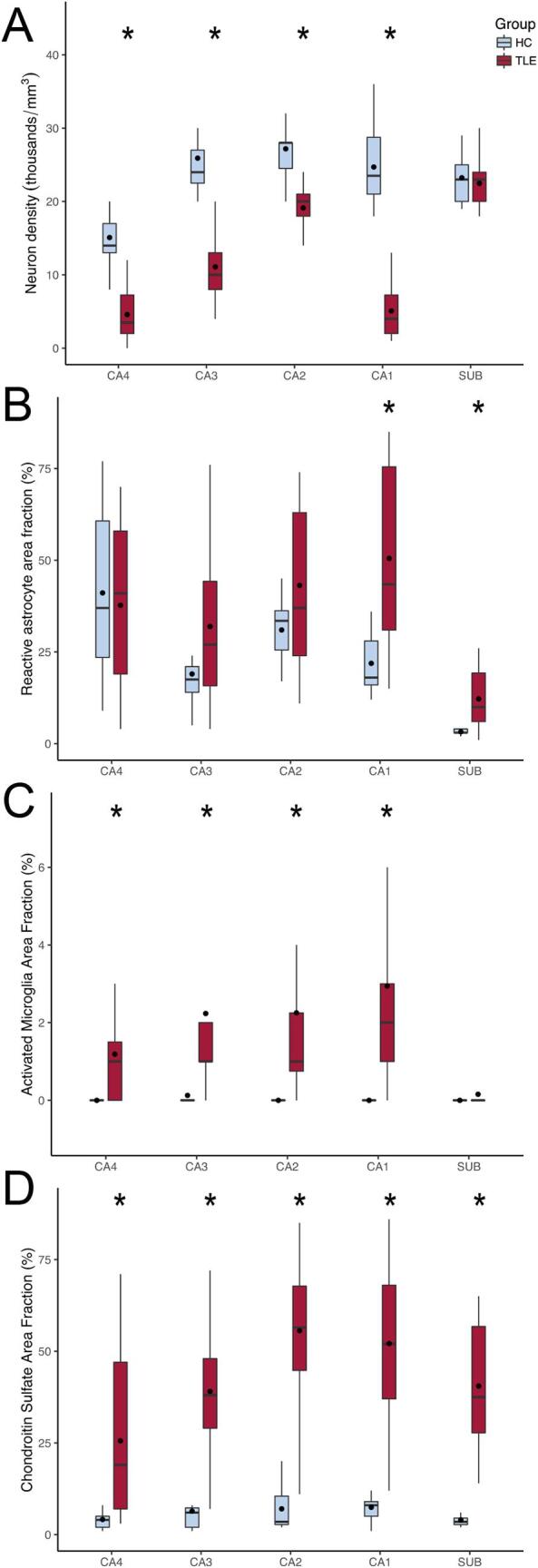
Semi-quantitative evaluation of hippocampal sections from controls (histological controls, blue boxplots) and TLE patients (red boxplots) submitted to immunohistochemistry. (A) All hippocampal subfields but the subiculum of TLE cases presented with neuron loss. (B) Only CA1 and the subiculum of TLE had significant astroglial reaction, when compared to controls. (C) Following the neuron density changes, all hippocampal subfields but the subiculum had activated microglia, when compared to controls. (D) Chondroitin sulfate proteoglycan was seen in higher levels in all hippocampal subfields of TLE cases, compared to controls. The asterisks indicate statistical difference, the line inside the boxplots indicate median and the dot indicate mean. (For interpretation of the references to colour in this figure legend, the reader is referred to the web version of this article.) (For interpretation of the references to colour in this figure legend, the reader is referred to the web version of this article.)

**Fig. 3:**
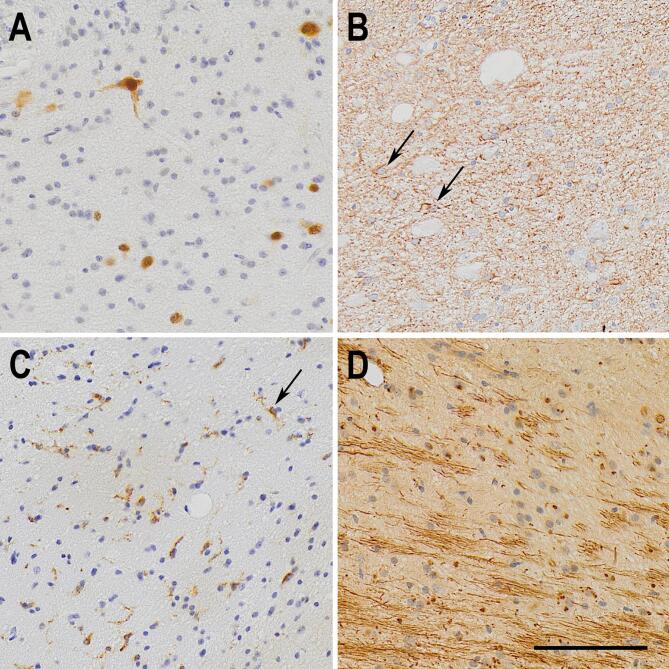
Representative immunohistochemistry micrographs of the pathological changes seen in the CA1 subfield from TLE samples. (A) NeuN staining, showing reduced neuron density in TLE CA1. (B) GFAP staining showing fibrous astrogliosis, as defined by ILAE, in a TLE case. Two reactive astrocytes are indicated by arrows. (C) Increased activation of microglial cells, marked by the expression of MHC class II HLA-DR protein. One activated microglia is pointed by the black arrow. (D) Fibrous aggregates of extracellular chondroitin sulfate proteoglycans, showed by CS-56 antigen immunohistochemistry. The bar in (D) indicates 100 µm.

TLE patients had lower neuron density than controls in CA4, CA3, CA2, and CA1 (Mann-Witney, p < 0.001), with no difference in the subiculum (Student, p = 0.629; Fig. 2 **A**). Reactive astrogliosis was seen in CA1 (Mann-Whitney, p = 0.002) and in the subiculum (Mann-Whitney, p = 0.003; Fig. 2 **B**) of TLE cases, compared to controls. Activated microglia was present in CA4 (Mann-Whitney, p = 0.016), CA3 (Mann-Whitney, p = 0.006), CA2 (Mann-Whitney, p = 0.005), and in CA1 (Mann-Whitney, p = 0.001; Fig. 2 **C**) of TLE patients, compared to controls. Increased expression of chondroitin sulfate proteoglycan was seen in CA4 (Mann-Whitney, p = 0.002), CA3 (Student, p < 0.001), CA2 (Mann-Whitney, p < 0.001), CA1 (Mann-Whitney, p < 0.001), and in the subiculum (Mann-Whitney, p < 0.001; Fig. 2 **D**). A representative image showing the expression patterns in TLE is shown in Fig. 3.

### 3.4 Associations between MRI, clinical data, and histology

Hippocampal MTR were not significantly different regarding HS type, the presence of focal to bilateral tonic-clonic seizures, the occurrence of status epilepticus or seizures in clusters, a positive familial history of seizures, seizure frequency, occurrence of IPI, or surgical outcome (see Table 2). Additionally, all three HS type 2 had normal hippocampal volume, two had increased T2 relaxation time, and one had lower MTR (Table 1). Logistic regression with the MTR classification (low vs. normal) reinforced the lack of association between MTR and the clinical data.

**Table 2:** MTR value according to clinical data classes.

Variable		Percentage of TLE cases	MTR	p-value
HS type	HS1	88%	48.314 (46.730–49.430)*	0.696
	HS2	12%	49.440 (47.927–50.740)	
Focal to bilateral tonic-clonic seizures	No	32%	48.460(47.625–50.675)	0.684
	Yes	68%	47.890(42.300–49.415)	
Seizure Clusters	No	20%	47.350(42.420–49.800)	0.706
	Yes	80%	48.810(47.460–49.430)	
Status Epilepticus	No	76%	48.810(47.460–50.225)	0.7.06
	Yes	24%	47.790(43.799–50.512)	
Familiar History of Seizures	No	42%	49.020(47.790–49.680)	0.743
	Yes	58%	48.387(46.182–49.690)	
Seizure Frequency	Daily	12%	49.430(48.460–50.880)	0.706
	Weekly	56%	47.998(46.317–49.500)	
	Biweekly	12%	47.460(46.730–50.470)	
	Monthly	20%	49.020(44.867–50.512)	
IPI Presence	No	54%	47.858(44.425–49.430)	0.684
	Yes	46%	49.112(47.935–50.273)	
Outcome	ILAE1	54%	48.635(47.673–49.877)	0.706
	ILAE2	14%	47.260(45.087–49.616)	
	ILAE3/4	32%	48.660(43.590–49.438)	

* Median (lower quartile – upper quartile).

**Fig. 4:**
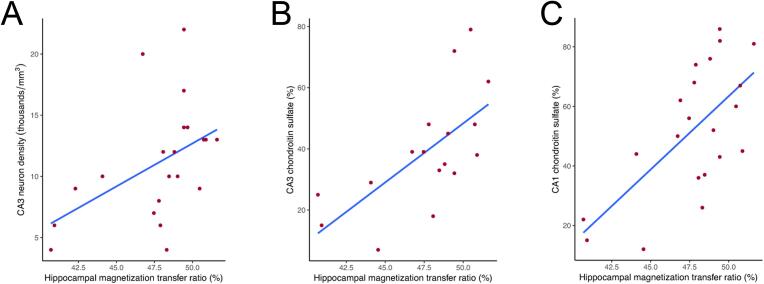
Scatterplot and regression between histological and magnetization transfer data of TLE patients. Whole hippocampal MTR from TLE cases correlated positively with neuron density in CA3 (A) and with chondroitin sulfate levels in CA3 (B), CA1 (C), and the subiculum (D).

Hippocampal MTR correlated positively with neuron density in CA3 (R = 0.581, p = 0.038; Fig. 4 **A**) and with CSPG levels in CA3 (R = 0.66, p = 0.038; Fig. 4 **B**) and CA1 (R = 0.544, p = 0.043; Fig. 4 **C**). Negative correlations between hippocampal MTR and activated microglia were seen in CA2 (R = -0.636, p = 0.043), and in CA1 (R = − 0.604, p = 0.038). Reactive astrocytes had no correlation with MTR.

From different combinations of neuron density and CSPG levels in CA4, CA3, CA1, and the subiculum, the best multiple linear model for explaining the variation in hippocampal MTR were the combination of neuron density and CSPG in CA3 and CA1, with a overall predictive value of 71% for the MTR (R = 0.841, R^2^ = 0.708, VIF < 2.971, p = 0.006). The following equation was given for these variables:

MTR = 40.282 + (0.324 × neuron density in CA3) - (0.274 × neuron density in CA1) + (0.0750 × CSPG levels in CA3) + (0.0429 × CSPG levels in CA1)

Individually, the changes in CA3 explain 59% of the hippocampal MTR (R = 0.767, R^2^ = 0.588, VIF = 1.049p = 0.003), whereas CA1 explains 43% of MTR (R = 0.652, R^2^ = 0.426, VIF = 1.035, p = 0.009). The multiple linear regression equations are as follows:

MTR = 39.779 + (0.349 × neuron density in CA3) + (0.100 × CSPG levels in CA3)

MTR = 43.487 - (0.131 × neuron density in CA1) + (0.0943 × CSPG levels in CA1)

## 4 Discussion

Reduced hippocampal volume and increased T2-weighted signal are important hallmarks of the epileptogenic hippocampi in drug-resistant TLE patients (Berkovic et al., 1991, Briellmann et al., 2002, Cendes et al., 1993). However, some patients have no MRI abnormalities in the standard MRI sequences (Bernasconi et al., 2000, Jackson et al., 1994). In these cases, new MRI techniques could help improve the MRI detection of the epileptogenic focus. Our present study evaluated MTR in the hippocampus of drug-resistant TLE patients and different hippocampal volumes on MRI, showing reduced MTR in the epileptogenic hippocampi. Half of the patients whose hippocampus had a normal volume on quantitative evaluation presented a reduced MTR, indicating that in normal volume cases MTR could help define the epileptogenic hippocampus. Moreover, in two cases (8% of our TLE) with normal volume and normal T2 relaxation, MTR was below normal levels. However, if an approach closer to the standard visual assessment (i.e., the visual asymmetry between ipsilateral and contralateral) is taken into account to define the epileptogenic hippocampus, MTR was by far the worse technique to indicate the epileptogenic hippocampus. Especially if we take into account that the very subtle differences in MTR are likely not detected by visual assessment, this technique is more interesting for research only. Moreover, lower MTR was not associated with any of the clinical variables investigated. Thus, our finding agrees with previous data in which reduced MTR was seen in the hippocampus related to seizure focus (Tofts et al., 1995), but indicates that MTR has very limited use for the lateralization of the focus in TLE cases with hippocampal sclerosis.

The atrophy seen in the epileptogenic hippocampus was linked to the degree of neuron loss in the granule cell layer (Briellmann et al., 2002) but more consistently to neuron loss in the CA1 subfield (Van Paesschen et al., 1997, Diehl et al., 2002). A previous study from our group also pointed out neuron loss in CA1, together with chondroitin sulfate levels in this subfield, as the most important factor for explaining hippocampal volume (Peixoto-Santos et al., 2015). Moreover, this subfield is, together with the subiculum, the largest hippocampal subfield (Peixoto-Santos et al., 2018), and is the most affected both in patients and in animal models (Blumcke et al., 2013, Do Val-da Silva et al., 2016). Reduced magnetization transfer has been linked to both myelin and neuron (axonal) loss in multiple sclerosis (van Waesberghe et al., 1999, Fisher et al., 2007, Mottershead et al., 2003). An animal model of autoimmune encephalomyelitis further indicated that MTR reduction in regions of neuron loss is more evident than in those with only demyelination (Rausch et al., 2009). In TLE, neuron loss is believed to be associated with MTR reduction (Flugel et al., 2006). However, a study with temporal cortex showed no correlation between neuronal population and MTR values (Eriksson et al., 2007). Our data indicate that neuron loss in CA3 is important for MTR reduction seen in TLE.

From the glial population evaluated with immunohistochemistry, only the activated microglia correlated with the hippocampal MTR. The glial reaction is a common finding in the hippocampus of drug-resistant TLE patients (Peixoto-Santos et al., 2015, Peixoto-Santos et al., 2017, Kandratavicius et al., 2015). Gliosis often following the degree of neuronal loss and seizure severity (Crespel et al., 2002, Khurgel and Ivy, 1996). However, the glial reaction is known to occur in the absence of neuron loss (Thom, 2014). Increased T2 signal and relaxation time is often linked to reactive astrogliosis (Van Paesschen et al., 1997, Briellmann et al., 2002), whereas no MRI sequence so far has been directly linked to microglia (Banati et al., 1999, Doorduin et al., 2009, Kumar et al., 2008). We saw negative correlations between MTR and activated microglia, but not with reactive astrocytes. Given the usually low increase in activated microglia in the hippocampus when compared to the often exuberant neuron loss and astroglial reaction, it would be unexpected for microglia to affect MRI sequences. It is possible that the inverse correlation between microglia and MTR is an indirect effect from the stronger association between neuron loss and CSPG with MTR. In fact, when added to the multiple linear regression model that included CA3 and CA1 values, the activated microglia presents the highest VIF values (2.935 for CA3 and 4.037 for CA1), indicating multicollinearity between microglia and other factors. Thus, we believe activated microglia should be seen as an indirect indicator for MTR changes.

ECM in the central nervous system is often studied concerning tissue plasticity (Silver and Miller, 2004, Huang et al., 2006). Increased CSPG levels were already described in patients with epilepsy and animal models (Perosa et al., 2002, Schwarzacher et al., 2006). In the pathology/MRI correlation field, some studies have shown an association between apparent diffusion coefficient changes and increased levels of CSPG (Roitbak and Sykova, 1999, Vorisek et al., 2002). Given that ECM accounts for 20% of the brain parenchyma (Davson and Spaziani, 1959, Sykova, 2004), it is expected that these molecules are important for MRI changes. In fact, previous studies of our group with hippocampi of TLE patients have shown a significant association between CSPG and changes in hippocampal volume and T2 relaxation (Peixoto-Santos et al., 2015, Peixoto-Santos et al., 2017). The link between ECM molecules and magnetization transfer is underexplored, and studies have provided mixed results. In phantoms of collagen and CSPG, while a study showed no correlation CSPG and MTR (Seo et al., 1996), another one has shown a weak positive association between CSPG and MTR (Laurent et al., 2001). Studies with animal cartilage also indicated that MTR is more often affected by the amount of collagen, with little effect of CSPG (Laurent et al., 2001, Toffanin et al., 2001). Our present data indicate that hippocampal MTR is strongly associated with the levels of CSPG. Since CSPG are increased in HS and correlate directly with MTR, one should expect an increased, and not decreased MTR in HS. However, neuron density is also directly proportional to MTR and is often lower in TLE. Thus, while neuron loss reduces MTR, increased CSPG increase it, and the balance between both will define if MTR will be within a normal range or significantly decrease. This could explain why some cases with HS type 1, the more severe HS in terms of neuron loss, have average MTR: in such cases, the increased CSPG expression may hide the intensity of neuron loss. A similar effect was seen in a previous study with another subset of patients, where some patients with severe neuron loss presented an average hippocampal volume due to a higher content of CSPG in the ECM (Peixoto-Santos et al., 2015). In summary, the presence of normal hippocampal MTR should not rule out the occurrence of hippocampal neuron loss and, thus, hippocampal sclerosis.

The most important limitation of the present study is, in our view, that we only evaluated the extracellular matrix with a broad-spectrum anti-chondroitin sulfate antibody. Besides the chondroitin sulfate proteoglycans, hyaluronic acid is a major constituent of the ECM (Perosa et al., 2002, Laurent and Fraser, 1992). Given that MTR is affected by macromolecules (Kim et al., 2014), and those ECM are some of the largest molecules in the brain, a more thorough evaluation of other extracellular molecules, such as phosphacan (the molecules that compose the perineuronal nets) and neurocan, as well as hyaluronic acid, should increase the correlation between ECM and MTR. However, ECM molecules are better evaluated with western blot, which was not feasible for our paraffin-embedded samples. An investigation with a more extensive series, especially with more HS type 2 and 3 patients, should evaluate the possible importance of MTR in separating HS types. These HS types are usually not distinguishable in presurgical MRI but can be identified in high-field ex-vivo MRI with smaller voxel size (Peixoto-Santos et al., 2018). Even if more HS type 1 cases where added, we believe stronger associations would emerge. Apart from HS types, we know that even the same HS type can had differential synaptic reorganization in several regions (Dombroski et al., 2020). It would be interesting to see if these, changes could impact MTR in higher field MRI, with smaller voxel sizes and subfield MTR assessment. In our consecutive sections from the hippocampal body immunostained for NeuN, GFAP, HLA-DR, and CSPG, we saw the same type of HS. However, we are aware that some studies indicated a variability of HS type across the hippocampal long axis (Steve et al., 2020, Thom et al., 2012). Although only a few studies have shown this variability, the use of several consecutive slices for the immunohistochemistry evaluation would also be necessary. However, we only had access to slices at the hippocampal body. Another critical problem of the MRI-histology correlational studies that is often not discussed is the problem of associating variables of different dimensionalities (i.e., onemm-thick MRI signal of whole hippocampal data with eight-µm-thin slices of immunohistochemistry evaluated in ROIs of different subfields). If replaced by whole-hippocampus western blot, we would lose subfields variability, but the general picture would be closer to whole hippocampal MTR values. The problem of comparing variables of different dimensions, as far as we know, has no clear, widely accepted solution. A better approach for future studies could be through immunohistochemistry evaluation of the hippocampal subfields on several slices throughout the hippocampus long axis and compare it with discrete MTR values of each subfield, and enrich the data with molecular approaches such as western blot and proteomics. Higher-field MRI with smaller voxels could also improve correlational studies.

## 5 Conclusions

The present study indicates that neuron density and extracellular matrix chondroitin sulfate are associated with MTR changes in the hippocampi of TLE patients, and together can explain more than 70% of the MTR signal in the ipsilateral hippocampus.

## Funding

This research was funded by Fundacao de Amaparo a Pesquisa do Estado de Sao Paulo (FAPESP), grant numbers 2015/20840–9 and 2016/17882–4.
